# The Lund University Checklist for Incipient Exhaustion: a prospective validation of the onset of sustained stress and exhaustion warnings

**DOI:** 10.1186/s12889-016-3720-7

**Published:** 2016-09-29

**Authors:** Kai Österberg, Roger Persson, Njördur Viborg, Peter Jönsson, Artur Tenenbaum

**Affiliations:** 1 Department of Psychology, Lund University, SE-22100 Lund, Sweden; 2 Department of Laboratory Medicine, Division of Occupational and Environmental Medicine, Lund University, SE-22185 Lund, Sweden; 3 School of Education and Environment, Centre for Psychology, Kristianstad University, SE-29188 Kristianstad, Sweden; 4 Hälsan & Arbetslivet, Occupational Health Care Unit, Region Västra Götaland, Skaraborg Hospital, SE-54185 Skövde, Sweden; 5 Department of Public Health and Community Medicine, Institute of Medicine, The Sahlgrenska Academy, University of Gothenburg, SE-40530 Gothenburg, Sweden

**Keywords:** Burnout, Exhaustion disorder, KEDS, LUCIE, Personality traits, Stress

## Abstract

**Background:**

The need for instruments that can assist in detecting the prodromal stages of stress-related exhaustion has been acknowledged. The aim of the present study was to evaluate whether the Lund University Checklist for Incipient Exhaustion (LUCIE) could accurately and prospectively detect the onset of incipient exhaustion and to what extent work stressor exposure and private burdens were associated with increasing LUCIE scores.

**Methods:**

Using surveys, 1355 employees were followed for 11 quarters. Participants with prospectively elevated LUCIE scores were targeted by three algorithms entailing 4 quarters: (1) abrupt onset to a sustained Stress Warning (*n* = 18), (2) gradual onset to a sustained Stress Warning (*n* = 42), and (3) sustained Exhaustion Warning (*n* = 36). The targeted participants’ survey reports on changes in work situation and private life during the fulfillment of any algorithm criteria were analyzed, together with the interview data. Participants untargeted by the algorithms constituted a control group (*n* = 745).

**Results:**

Eighty-seven percent of participants fulfilling any LUCIE algorithm criteria (LUCIE indication cases) rated a negative change in their work situation during the 4 quarters, compared to 48 % of controls. Ratings of negative changes in private life were also more common in the LUCIE indication groups than among controls (58 % vs. 29 %), but free-text commentaries revealed that almost half of the ratings in the LUCIE indication groups were due to work-to-family conflicts and health problems caused by excessive workload, assigned more properly to *work-related* negative changes. When excluding the themes related to work-stress-related private life compromises, negative private life changes in the LUCIE indication groups dropped from 58 to 32 %, while only a negligible drop from 29 to 26 % was observed among controls. In retrospective interviews, 79 % of the LUCIE indication participants confirmed exclusively/predominantly work stressors, while 6 % described a predominance of private life stressors.

**Conclusions:**

Negative changes in the work situation were the most prominent change related to a sustained increase in LUCIE scores. The findings seem to confirm that LUCIE is a potentially useful tool for clinical screening of incipient work-related exhaustion.

**Electronic supplementary material:**

The online version of this article (doi:10.1186/s12889-016-3720-7) contains supplementary material, which is available to authorized users.

## Background

Psychiatric illness and sick leave due to work-related stress are acknowledged major public health concerns in many postindustrial societies [1]. Being primarily manifested via the ICD-10 (International Statistical Classification of Diseases and Related Health Problems, 10th Revision) categories depressive episode (F32) or reaction to severe stress (F43), sick leave due to psychiatric conditions related to work is often of long duration and imposes a heightened risk of early retirement [1–3]. In Sweden, the proportion of long-term sickness absence (>60 days) due to psychiatric diagnoses escalated sharply from 18 % in 1999 to 30 % in 2003 [4]. After a decline between 2005 and 2009, a recent period of increasing levels followed, and in 2014 psychiatric diagnoses were estimated to constitute 35 % of all long-term sickness absences (38.5 % for women and 27.5 % for men) [4].

In 2003, steps were taken within the health care system to improve the management of the escalating signs of mental health problems in the working population. To clarify the diagnostic practices, the Swedish National Board of Health and Welfare (NBHW) suggested a set of criteria to establish a new diagnostic entity termed Exhaustion Disorder (ED) [5]. Accordingly, in 2005, the NBHW introduced ED as an additional specification within the ICD-10 category F43.8 (other reactions to severe stress). In brief, the ED diagnosis requires a clearly identifiable stress exposure of at least 6 months’ duration combined with markedly reduced mental energy and other similar symptoms as well as significant impairment in everyday social and occupational functioning. Compared with the burnout construct, which centers on psychological manifestations of exhaustion [6], ED is more physiologically oriented. Beyond exhaustion, ED entails a reduced activity level, an increased need for recovery and diverse symptoms (e.g., pain, impaired memory, insomnia) that cause distress in social and/or work life.

Because the negative consequences of stressor exposures that last for several months or years often lead to long sick leaves and complicated rehabilitation procedures, interest in and efforts toward detecting *prodromal* stages of ED have increased. Indeed, both clinical experience and research indicate that fairly simple and brief intervention techniques suffice to reverse the development toward ED if such techniques are applied in the early stages [7, 8]. Drawing on this insight, and to meet the need for early detection instruments, we developed the Lund University Checklist for Incipient Exhaustion (LUCIE). LUCIE’s thematic content and selection of items were driven and inspired by clinical interviews held with around 100 patients with confirmed work-related exhaustion who were participating in a workplace intervention study [9, 10]. Specifically, LUCIE takes a syndrome approach and aims to detect early manifestations of signs of stress and exhaustion across six domains: sleep and recovery, separation between work and spare time, sense of community and support at the workplace, managing work duties and personal capabilities, private life and spare time activities, and health complaints. A cross-sectional validation, which entailed comparing LUCIE with other contemporary tools for detecting ED, has recently been presented by Persson et al. [11].

In the present 3-year longitudinal study, the focus was on evaluating whether LUCIE could accurately detect individuals developing incipient exhaustion. After prospectively identifying participants who showed signs of incipient exhaustion in LUCIE, we examined their reports of changes in work situation and private life circumstances during the period of time leading up to the subsequent signs of exhaustion in LUCIE. Because LUCIE was developed in working populations with workload as one criterion for item selection, our main hypothesis was that signs of incipient exhaustion should be primarily associated with a perceived increase in work stress.

## Methods

### Study design

The present paper reports on a 3-year longitudinal study including one baseline survey (T0) and 10 equally spaced (i.e., at 3-month intervals) consecutive surveys (T1 to T10) as well as telephone interviews. The baseline survey was issued in spring 2012 (T0) and was returned by mail together with a completed informed consent form. The subsequent 10 surveys were completed online using the software Textalk Websurvey (www.textalk.se; Gothenburg, Sweden). The second survey (T1) was issued the first week of September 2012. The data were thereafter collected in December, March, June, and September until December 2014 (T10). Each websurvey had a response window of circa three weeks (or slightly longer in case of interfering public holidays), during which a maximum of three e-mail reminders were issued to non-responders. Telephone interviews served as a supplement to the surveys for participants with prospectively identified sustained stress or exhaustion warning in LUCIE (for details see section *Interview data* below).

### Participants

In total, 1355 participants from southern Sweden were eligible to be prospectively followed over the 3-year study period. The recruitment process was described in detail by Persson et al. [11], and a brief summary follows. A total of 7799 persons, who had either been respondents to a population survey [12] or identified through the population registry of Skåne University Hospital in southern Sweden, were invited by letter to participate. The invitation letter stated that participants were expected to be gainfully employed at least 75 % of full-time, not to have had any period of long-term full-time sick leave for the past six months, nor to have had a chronic disease or been on daily medication. In total, 1400 persons (18 %) returned a completed baseline questionnaire together with a completed informed consent sheet and were included as potential participants. Data from the baseline questionnaire were used to refine the selection of participants. Exclusion criteria were self-reports of somatic disease, daily medication with psychotropic drugs, excessive alcohol consumption, part-time work below 75 % of full time (<30 h/week), and recent longer full-time sick leave. Forty-five persons failed to pass these criteria and the final cohort consisted of 1355 participants (57 % women). Their mean age at baseline was 41.1 years (SD 6.7 years; range 27–52). University education was reported by 67 %, secondary school by 32 % and elementary school by less than 1 %. Full-time occupational activity (40 h/week) was reported by 83 %. The vast majority were salaried employees, and 10 % were either self-employed or combined paid employment with self-employment [11].

### Measures

#### Questionnaire data

*Lund University Checklist for Incipient Exhaustion (LUCIE)* consists of 28 items describing behaviors and feelings associated with the prodromal stages of exhaustion disorder. LUCIE is intended to be a tool for identifying the prodromal stages of work-stress-related exhaustion and is based on qualitative analyses of ED patients’ interviews/narratives concerning their earliest signs of ED. (See Persson et al. [11] for a detailed description of the basic development of LUCIE and item contents). In LUCIE, the instruction to the respondent is: “For the past month, to what extent have you felt or observed the following?” The response to each item is made on a 4-point scale: 1 = not at all, 2 = somewhat, 3 = quite a bit, and 4 = very much. The LUCIE items cover six domains: (a) sleep and recovery, (b) separation between work and spare time, (c) sense of community and support in the workplace, (d) managing work duties and personal capabilities, (e) private life and spare time activities, and (f) health complaints.

The detection of incipient exhaustion in LUCIE builds on two algorithms comprising two separate, supplementary scales: the *Stress Warning Scale (SWS),* which is sensitive to milder signs of incipient exhaustion, and the *Exhaustion Warning Scale (EWS),* which is intended to reflect more severe signs of exhaustion*.* The general difference between the SWS and the EWS algorithms concerns the *intensity* of the replies, as the EWS score is based mainly on replies at the highest level (“very much”), while replies on the next lower level (“quite a bit”) are also included in computation of the SWS. A wide range of stress signs on the next lower level is thus only reflected in a high SWS score, while the extent of replies on the highest level are recorded on the EWS scale. The purpose with this division on two scales is to enable the clinician to easily assess whether the LUCIE result indicates a slight to moderate state of stress (high SWS and low EWS) or if signs are so intense that ED might be suspected (high EWS) [11]. The SWS and EWS computation algorithms are presented in detail in Additional file 1.

The scores on both scales range from 0 to 100. A low SWS score (≤ 17.00; ‘the green zone’) is intended to indicate normal/negligible long-term stress symptoms. A slightly higher SWS score (between 17.01 and 38.50; ‘the yellow zone’) suggests possible slight stress symptoms. A rather high SWS score (≥38.51; ‘the red zone’) indicates mild to moderate stress symptoms. When the SWS score reaches the red zone, it is recommended to start checking the EWS score for more severe symptoms of stress, possibly indicating exhaustion. A low EWS score (≤ 21.50; ‘the EWS green zone’) indicates that signs of exhaustion are mostly absent or mild, while a higher score (> 21.50; ‘the EWS red zone’) suggests severe symptoms that might indicate exhaustion disorder, in that case overriding any SWS score. In practice, the combined scores on the SWS and the EWS provide a 4-step severity ladder of stress symptomatology:

1. *Step 1-GG* (SWS green zone and EWS green zone) = no or negligible lasting stress symptoms
2. *Step 2-YG* (SWS yellow zone and EWS green zone) = possible slight lasting stress symptoms
3. *Step 3-RG* (SWS red zone and EWS green zone) = mild to moderate lasting stress symptoms, but less severe than ED
4. *Step 4-RR* (SWS red zone and EWS red zone) = lasting stress symptoms of a severity indicating possible ED.

Because the other theoretically plausible combinations of scores (i.e., SWS green zone or SWS yellow zone in combination with EWS red zone score) are extremely rare in clinical settings and in population samples [11], the four ranking steps above are practical simplifications. In cases where a high SWS score is observed, the supplementary EWS measure will indicate whether the stress symptomatology is of an intensity indicative of ED (Step 4-RR), or is more benign in nature (Step 3-RG).

*Changes in the situation at work and in private life* were assessed using two newly constructed items that addressed perceived positive or negative changes in (a) the work situation or (b) private life. The items read: “Has your situation at work changed in a positive or negative direction during the past couple of months?” and “Has the situation in your private life changed in a positive or negative direction during the past couple of months?” Responses to both items were made on a 5-point scale: 1 = Yes, in a highly positive direction, 2 = Yes, to some extent in a positive direction, 3 = No, no significant change, 4 = Yes, to some extent in a negative direction, 5 = Yes, in a highly negative direction. As a supplement, participants were also encouraged to fill in an optional text field (480 signs) with free-text commentaries.

*The Karolinska Exhaustion Disorder Scale* (KEDS) was used to validate that a prospective elevation of LUCIE scores (see *Identification of cases* below) reflected genuine signs of exhaustion. KEDS is a recently developed tool for screening for the presence of ED; it contains nine items selected to correspond with the ED criteria specified by the NBHW in 2003 [13]. The item contents are: (1) ability to concentrate, (2) memory, (3) physical stamina, (4) mental stamina, (5) recovery, (6) sleep, (7) hypersensitivity to sensory impressions, (8) experience of demands, and (9) irritation and anger. Each item has seven response alternatives, ranging from 0 to 6, with higher values reflecting more severe symptoms. The sum of item scores constitutes the outcome (range 0 – 54). A sum of item scores ≥ 19 is the recommended cutoff criterion for ED, which was shown to optimize both sensitivity and specificity [13].

*Personality traits* were assessed at baseline (T0) with a Swedish version of the 44-item Big Five Inventory (BFI), which includes the dimensions: Neuroticism, Extraversion, Openness, Agreeableness and Conscientiousness [14, 15]. The items of the BFI are short and easily understandable phrases, and each BFI item is rated on a 5-point scale with verbal labels ranging from “Disagree strongly,” (score 1), “Disagree a little,” “Neither agree nor disagree,” “Agree a little,” and “Agree strongly” (score 5). Each of the Big Five personality dimensions was calculated as the mean score of the 8–10 items covering the dimension.

#### Interview data

Telephone interviews were carried out to collect data on the substance of LUCIE indications and the perceived sources of stress. Specifically, participants showing a prospective elevation of LUCIE scores (see *Identification of cases* below) were informed by letter that unspecified changes in their questionnaire replies had been observed and told we would contact them by telephone for a brief interview. The interviews were carried out by an experienced clinical psychologist and psychotherapist (N.V.). When we reached the participant by phone, he/she was informed that we had seen, during the past two quarters, increased ratings on a set of questions that *might* be related to work stress. The interviewer then asked the participant the following two questions, one regarding work stressors and the other concerning stressors outside work (private life). The first question read “*In your opinion, have you experienced more stress at work lately (during the past six months) than previously and, if so, to what extent?*” The second question read “*Do you think there are other reasons (outside work) for the changes in your questionnaire replies and, if so, to what extent*?” In practice, these two questions were not given in verbatim, and were often conveniently combined into one question; “H*ave you experienced more stress at work during the past quarters, or do you think there are other reasons (outside work; e.g., private stress) for the changes in your questionnaire replies?”* The reply obtained within each area was scored by the interviewer as: 3 = a substantial increase; 2 = a moderate increase; 1 = a slight increase; 0 = no increase or a decrease. After the interview, the balance between reported work and private life stressors was scored as: 1 = work stressors only; 2 = predominantly work stressors but also some private life stressors; 3 = roughly equal shares of work stressors and private life stressors; 4 = predominantly private life stressors but also some work stressors; 5 = private life stressors only. Of the 56 participants we were able to reach by phone, 26 also accepted a clinical consultation with the psychotherapist, which provided a richer background which, however, only in a few cases led to minor adjustments in the interviewer ratings.

### Identification of cases with sustained stress or sustained exhaustion warning

Each participant was prospectively followed throughout the study period with the intent to identify the first onset of an episode exhibiting a *sustained stress warning* or a *sustained exhaustion warning* in LUCIE. A LUCIE indication case was defined by an episode of two consecutive scores in the red zone on the SWS or the EWS scale, preceded by more or less low/normal scores for the two previous quarters (except in the first sampling round at T2, see below). Once identified, the interview protocol was commenced (see above).

#### Algorithms for identification of sustained stress warning cases

1. *Abrupt onset to sustained stress warning (SWS-AS):* This algorithm identified cases that had two consecutive LUCIE SWS scores in the green zone (score ≤ 17.00) followed by two consecutive scores in the red zone (score > 38.50).
2. *Gradual onset to sustained stress warning (SWS-GS):* This algorithm identified cases that started with SWS scores in the green *or* yellow zone (≤ 38.50), followed by a score in the SWS yellow zone (>17.00 and ≤ 38.50), and then two consecutive scores in the SWS red zone (score > 38.50). To avoid targeting individuals with very minor increases in SWS around the cutoff border (e.g., an increase from SWS = 37 to SWS = 39), an additional criterion was that the SWS red zone scores should be at least 20 points higher than observed in the preceding ‘yellow zone’ phase.

#### Algorithms for identification of sustained exhaustion warning cases

3. *Onset to sustained exhaustion warning (EWS-S)*: Irrespective of SWS scores, this algorithm identified cases that had two consecutive EWS scores in the green zone (≤ 21.50) followed by two consecutive EWS scores in the red zone (> 21.50).

#### Modified algorithms in the first sampling round

In order to maximize the number of participants with a LUCIE indication, slightly simplified algorithms were used in the initial sampling round at T2. We targeted individuals already at T2 if their single baseline (T0) LUCIE score was normal, followed by elevated scores both at T1 and T2. The modified algorithms were as follows:

1. *Abrupt onset to sustained stress warning (SWS-AS):* A LUCIE SWS score in the green zone (score ≤ 17.00) followed by two consecutive scores in the red zone (score > 38.50).
2. *Gradual onset to sustained stress warning (SWS-GS):* A SWS score in the SWS yellow zone (>17.00 and ≤ 38.50) followed by two consecutive scores in the SWS red zone (score > 38.50), both being ≥ 20 points higher than the preceding yellow zone score.
3. *Onset to sustained exhaustion warning (EWS-S)*: An EWS score in the green zone (≤ 21.50) followed by two consecutive EWS scores in the red zone (> 21.50).

Participants fulfilling any LUCIE algorithm criteria are henceforth referred to as ‘LUCIE indication cases’, and the phrase ‘after onset of stress/exhaustion indication’ refers to the latter two quarters with elevated LUCIE scores.

### Data management

When four consecutive LUCIE scores, based on the algorithms given above, confirmed the onset of a sustained stress or exhaustion warning, the time for the fourth quarter was set to Q 0 (Q zero) and the preceding three quarters were defined as: Q minus 3, Q minus 2 and Q minus 1 (Q -3, Q -2, and Q -1). Thus, taking an event-based approach, we corralled all identified events into a new dataset consisting of four quarters (Q-3 to Q 0). Participants with no indication of a sustained stress or exhaustion warning during the study period (*n* = 1259), and with complete sets of data during the final four assessment waves (T7 to T10), constituted the control group (*n* = 745). For controls, the final websurvey (T10) was thus defined as Q 0. Of the 745 final controls, 82 % had responded to all 11 quarterly surveys, 17 % failed to reply on 1–3 quarters, and less than 1 % on ≥ 4 quarters. The subgroup of potential controls that did not have complete data during the final four quarters (*n* = 514) did not differ from the final control group concerning demographic data or self-reports concerning, for example, age, education, personality scores, or LUCIE scores (data not shown).

### Statistical analysis and analysis of free-text answers

*P*-values ≤ 0.05 (two-tailed) were considered statistically significant. Visual inspection of the data revealed that the LUCIE scores were positively skewed. The data from the single-item questions on changes in the situation at work and in private life were also considered unsuitable for parametrical analysis. However, the BFI data were approximately normally distributed. Thus, all data except BFI data were analyzed using non-parametrical tests. Between-groups analyses entailing categorical data were conducted using Pearson’s *χ*2-tests. Only BFI data were analyzed using one-way ANOVA with Post Hoc LSD tests, and effect sizes are given as partial eta square (*η*_p_^2^). All tests were made using SPSS/IBM software, version 22.

Thematic analyses of the free-text commentaries were made through an iterative process. To begin with, changes in the work/private situation were scrutinized to establish empirically common and broad general themes. Then a more fine-grained reading of each commentary sorted them into preliminary thematic categorizes. Next, changes in the work situation themes were, if possible, broadly categorized according to themes found in extant mainstream models of work stressors, e.g., the demand-control-support model, the effort-reward model and the organizational injustice model. Finally, an iterated check of the free-text commentaries was carried out to confirm or disconfirm the previous rounds of classification.

## Results

### Onset of sustained stress warning and sustained exhaustion warning

**Fig. 1:**
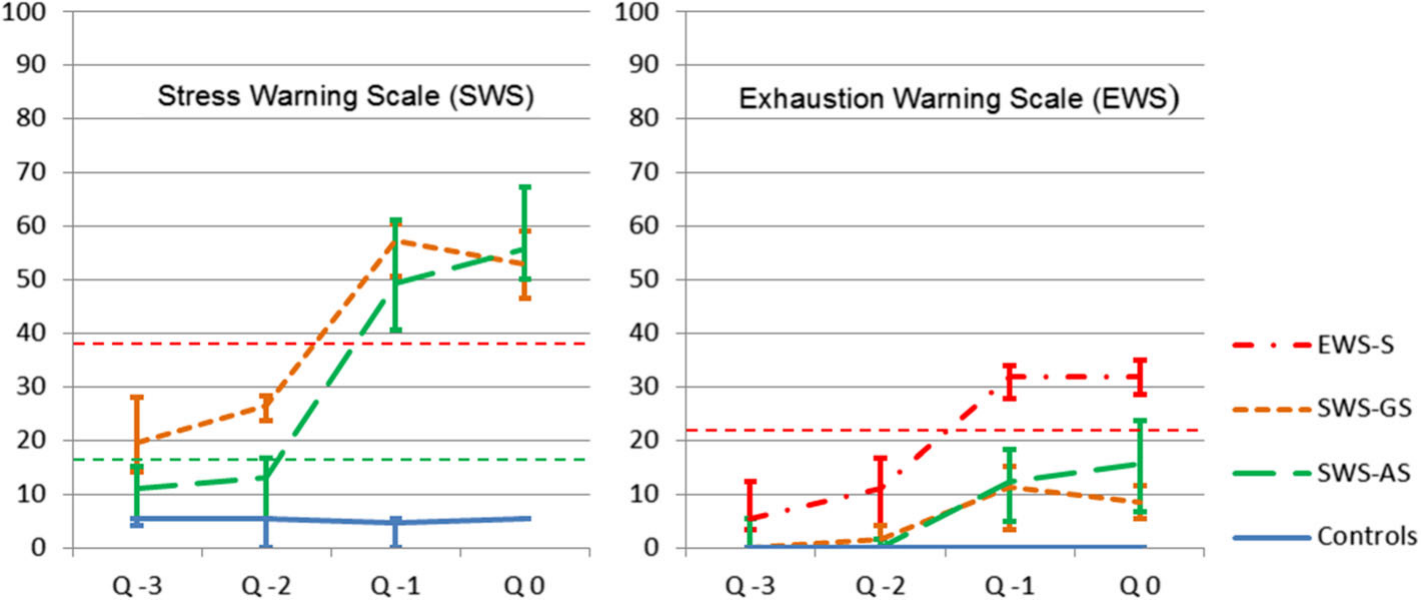
LUCIE median values (95 % CI) during four quarters. Left panel show SWS values for the subgroups with onset of stress warning (SWS-AS and SWS-GS) and controls, and right panel show EWS values for all subgroups. The *thin horizontal lines* (broken) show the borders between SWS-green/yellow/red zones (left panel) and between EWS-green/red zones (right panel)

In total, 96 individuals exhibited a sustained stress or exhaustion warning in LUCIE (the temporal dispersion was: T2: *n* = 23, T3: *n* = 15, T4: *n* = 15, T5: *n* = 13, T6: *n* = 3, T7: *n* = 9, T8: *n* = 4, T9: *n* = 7, and T10: *n* = 7). Of these, 18 persons had an abrupt onset to a sustained stress warning (SWS-AS), whereas 36 persons had a gradual onset (SWS-GS). The remaining 42 individuals had a sustained exhaustion warning (EWS-S). The median levels of SWS and EWS ratings in the subgroups are shown in Fig. 1. Most demographic parameters were fairly equally distributed in the LUCIE indication and control groups. A higher proportion of women displayed indications in LUCIE (Table 1).

**Table 1:** Baseline demographical characteristics of the participants identified as having a sustained stress or exhaustion warning in LUCIE and controls

Characteristic	LUCIE indication subtype			Any LUCIE indication (*n* = 96)	CONTROL (*n* = 745)
	SWS-AS (*n* = 18)	SWS-GS (*n* = 42)	EWS-S (*n* = 36)		
Age					
Mean (SD)	44.1 (5.1)	41.0 (7.1)	41.2 (6.0)	41.7 (6.4)	41.8 (6.5)
Range	32–51	28–52	31–50	28–52	27–52
Gender (%)					
Men	22	31	25	27	46
Women	78	69	75	73	54
Education (%)					
Nine-year compulsory schooling	0	0	0	0	0
Upper secondary school	39	28	28	26	30
University studies	61	72	72	74	70
Occupational activity (%)					
Full-time work (≥40 h/week)*	100	88	83	88	83
Part-time work (30–39 h/week)	0	12	17	12	17
Employment (%)					
Salaried employee	83	95	78	86	91
Self-employed	11	2	17	9	5
Combined self-employment and employee	6	2	6	4	4

*SWS-AS* Abrupt onset to sustained stress warning, *SWS-GS* Gradual onset to sustained stress warning, *EWS-S* Onset to sustained exhaustion warning

### Validation of exhaustion-related symptoms

**Fig. 2:**
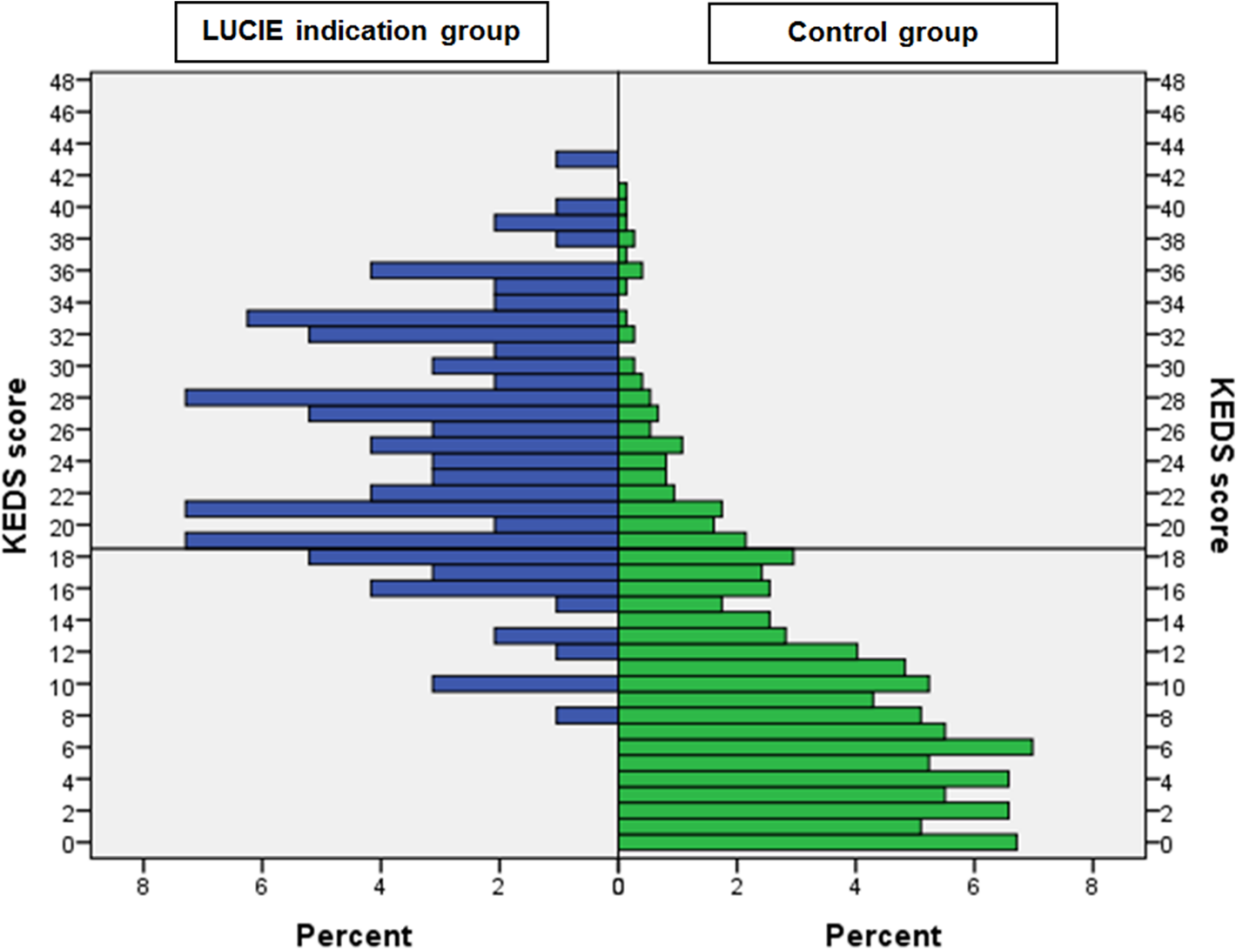
Distribution of KEDS scores at Q 0 among LUCIE indication cases (*n* = 96) and controls (*n* = 745). The *solid horizontal line* shows the cutoff score for ED indication (≥ 19)

At the time of fulfilling the sustained SWS or EWS criteria, 79 % of the LUCIE indication cases showed a KEDS score indicative of ED (i.e., a score of 19 or above), compared to 13 % among controls (*p* < 0.001; Pearson’s *χ*2-test). Within the LUCIE indication subgroups, a slightly higher rate was observed in the EWS-S group (86 %) than in the SWS-AS and SWS-GS groups (72 and 76 %, respectively), but the difference between distributions did not reach statistical significance (*p* = 0.4). However, the LUCIE indication cases with KEDS scores falling below the cutoff for ED indication also tended to have higher KEDS scores than most control participants; see Fig. 2.

### Changes in the work situation associated with onset of sustained stress warning and sustained exhaustion warning

#### Evaluation of the relative proportion of positive and negative changes in the work situation

**Fig. 3:**
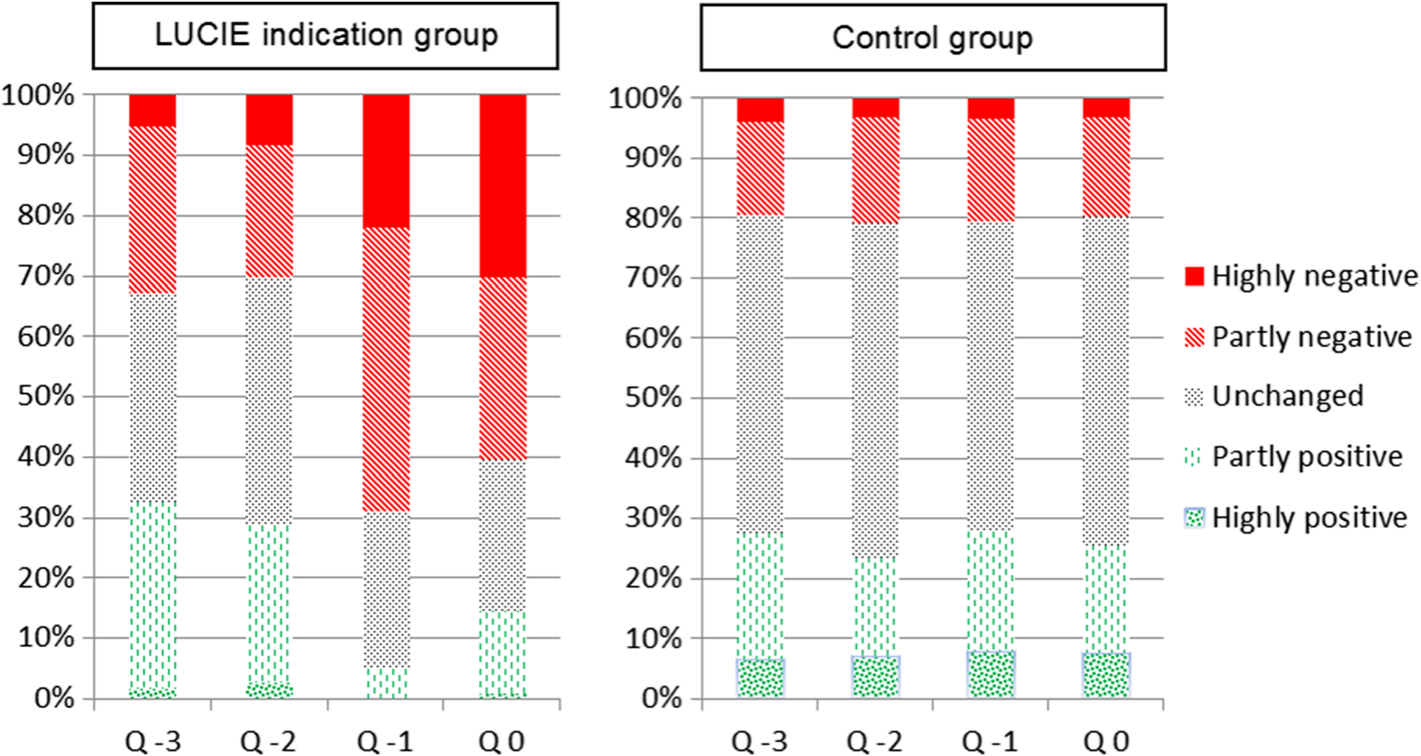
Ratings of changes in the work situation. Left panel shows ratings during the four quarters leading up to a sustained stress or exhaustion warning among LUCIE indication cases (*n* = 96), and right panel shows the corresponding data for controls (*n* = 745)

An overview of the ratings for each separate quarter is given in Fig. 3. Pearson’s *χ*2-tests indicated that ratings of negative changes in the work situation were more common among participants with sustained stress or exhaustion warning than among controls; 87 % of the LUCIE indication participants reported at least one negative change during the four quarters, versus 48 % of the controls (*p* < 0.001). The group difference was more pronounced after onset of indication, that is, in the final two quarters (82 and 32 %, respectively; *p* < 0.001). There was no overall difference between the SWS-AS (78 %), SWS-GS (83 %), and the EWS-S (94 %) groups across all four quarters (*p* = 0.18), nor during the final two quarters (*p* = 0.15). During the two quarters before onset, there was no difference between the LUCIE indications groups (33 %) and the controls (32 %) (*p* > 0.7).

Ratings of *positive* changes in the work situation during the four quarters were equally common among participants with a sustained stress or exhaustion warning as among controls (48 and 54 %, respectively; *p* = 0.23). There were no differences in ratings between the SWS-AS (56 %), SWS-GS (45 %), and the EWS-S (47 %) groups (*p* = 0.8). However, considerably fewer in the subgroups with a LUCIE indication, compared to controls, rated a positive change after onset of a sustained stress or exhaustion warning (18 % vs. 39 %; *p* < 0.001).

#### Analysis of free-text answers related to positive and negative changes at work

*Statements concerning negative changes*: Of the LUCIE indication participants (i.e., SWS-AS, SWS-GS, and EWS-S) who acknowledged a negative change in their work situation (*n* = 83), 77 provided a free-text description of the change. The proportions of free-text contributions in the three subgroups were similar in numbers and themes, which is why we collapsed them in the analyses. The qualitative thematic analysis identified 13 main categories of complaints (Additional file 2: Table S2:1). The most common category was *increased workload/demands, including shortage of staff* (57 %), followed by *reduced support from supervisor or colleagues* (23 %) and various *organizational problems or a negative organizational change* (13 %). Half of the participants (48 %) reported two or more negative categories during the four quarters.

*Statements concerning positive changes*: Among the 46 participants rating any positive change, 40 also gave free-text descriptions. The qualitative thematic analysis identified 11 main categories (Additional file 2: Table S2:2). The most common category was *enriched decision latitude or more exciting/stimulating work tasks* (30 %) and *improved support from supervisor, colleagues or through group intervention* (33 %), followed by *successful move to new employer* (*n* = 17 %) and *reduced workload and/or decreased emotional or intellectual demands from supervisors, including reduced shortage of staff* (17 %).

### Changes in the private situation associated with onset of sustained stress warning and sustained exhaustion warning

#### Evaluation of the relative proportion of positive and negative changes in the private sphere

**Fig. 4:**
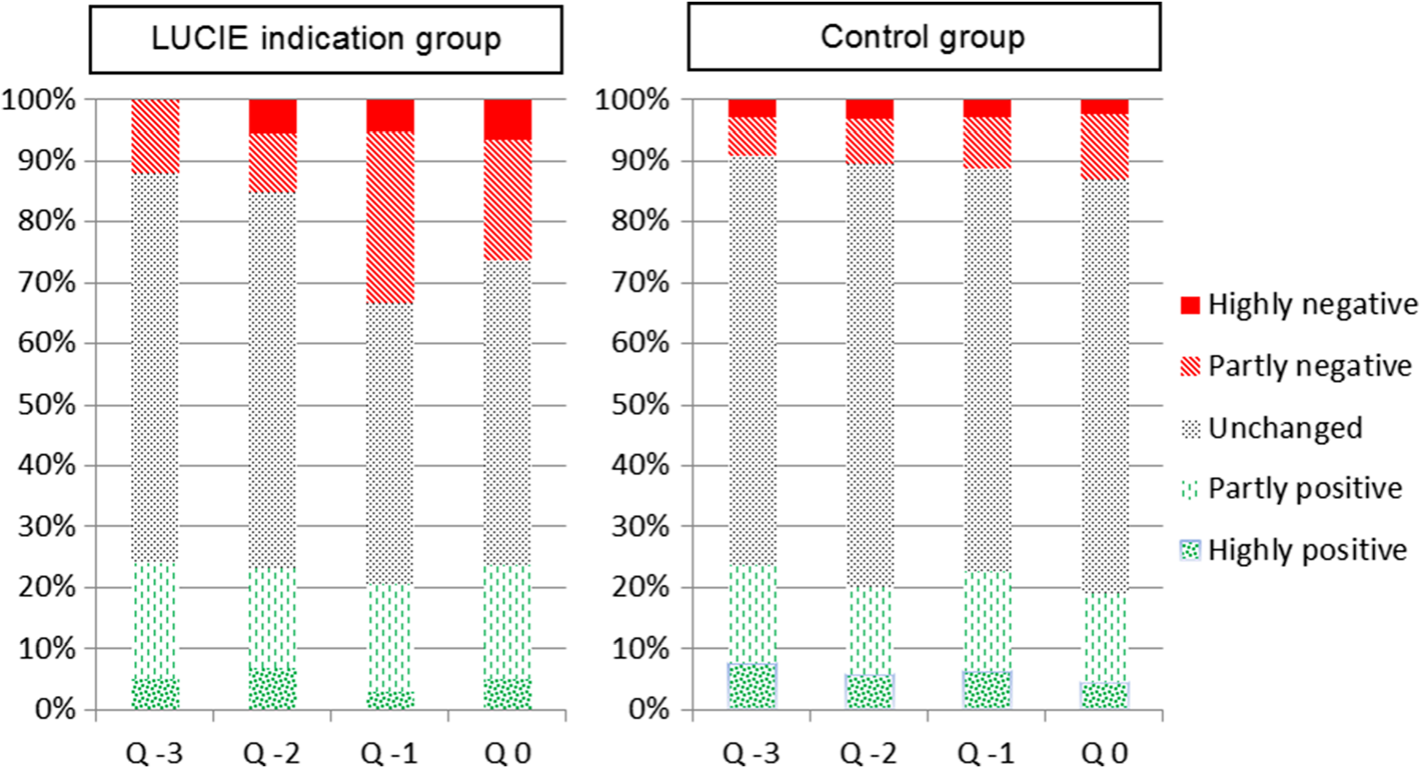
Ratings of changes in the private life situation. Left panel shows ratings during the four quarters leading up to a sustained stress or exhaustion warning among LUCIE indication cases (*n* = 96), and right panel shows the corresponding data for controls (*n* = 745)

An overview of the ratings for each separate quarter is given in Fig. 4. Pearson’s *χ*2-tests indicated that ratings of negative changes in the private situation at least once during the four quarters were more common among participants with a sustained stress or exhaustion warning than among controls (58 and 29 %, respectively; *p* < 0.001). The proportions of participants with negative ratings were similar in the SWS-AS (61 %), SWS-GS (60 %), and EWS-S (56 %) groups (*p* = 0.9). Prior to onset of stress/exhaustion warning, that is, the first and second of the four quarters, there was no difference between the LUCIE indications groups (33 %) and the controls (32 %) (*p* > 0.7).

Ratings of *positive* changes in the private situation did not differ between participants with sustained stress or exhaustion indication and controls (50 and 47 %, respectively) (*p* = 0.6). Likewise, there was no difference between groups after onset of stress/exhaustion indication (*p* = 0.9). Moreover, there was no difference between the proportions reporting positive change in the SWS-AS (44 %), SWS-GS (45 %) and EWS-S (58 %) groups (*p* = 0.4).

#### Analysis of free-text answers related to positive and negative changes in the private sphere

*﻿Statements concerning negative changes*: Among the 56 participants who had rated any negative change in private life during any of the four quarters, 46 also gave free-text descriptions. The qualitative thematic analysis identified 13 main categories (see Additional file 2: Table S2:3). The most common category by far was *work-family conflict (lack of time/energy)* (*N* = 22; 48 %). Other common categories were *feeling worn-out (fatigue, exhaustion)* (24 %) and *relational problems in family* (15 %).

*Statements concerning positive changes:* Among the 48 participants reporting a positive change in private life during any of the four quarters, 43 also gave free-text descriptions. The qualitative thematic analysis identified 13 main categories (Additional file 2: Table S2:4). *Improved family relations or family situation* was the most commonly reported positive change (37 %), but 15 % also reported a *voluntary reduction of work-hours in order to cope* with life and family. This means that reports of private life improvements in the LUCIE indication groups were to some extent inflated by intentional actions to reduce workload, which was in parallel reported as a positive change in the *work situation* – and *vice versa*.

#### Disentangling work-related burden from private circumstances

When analyzing the free-text statements of private life stressors, it became apparent that many participants with a LUCIE indication had reported negative impact from work on private life as “negative changes in private life.” This observation was analyzed in more detail. If we discount the acknowledgements of *work-to-family conflict (lack of time/energy)* from “classical” private burdens (i.e., relational problems in the family, death of relatives, problems with children, divorce, etc.), only 34 of the 46 respondents acknowledged a negative change. If we further discount acknowledgments of *feeling worn-out due to work (fatigue, exhaustion)*, only 25 of the 46 respondents (54 %) still maintained a traditional negative change in their private situation. If extrapolated to represent all 56 negative raters among the total of 96 LUCIE indication cases (54 %*56 = 30), this suggests that only 30 of the 96 LUCIE indication cases (32 %) would be likely to have a genuine private burden *unrelated* to work.

To explore the similarity with controls on this point, we selected a random sample of 100 controls from the 217 of the 745 controls who had rated a negative change in their private situation during at least one of the four quarters. Of these 100 controls, 88 had given free-text descriptions. If, again, we discount the themes *work-family conflict (lack of time/energy)* and *feeling worn-out due to work (fatigue, exhaustion)* from the reported “negative changes in private life,” 77 of the 88 respondents (87 %) still acknowledged a “traditional” negative change in their private situation (e.g., relational problems in family, death of close relatives; see Additional file 2: Table S2:3). Extrapolated to represent all 217 negative raters (87 %*217 = 190), this suggests that 190 of the 745 controls (26 %) would still be likely to have a genuine private burden *unrelated* to work.

Thus, if we subtract the negative impact of work on private life from the “negative changes in private life,” the net prevalence of “traditional” or “genuine” private burdens *unrelated* to work become rather similar in the LUCIE indications group (32 %) and among controls (26 %).

### Interview data

Of the 96 participants targeted by any LUCIE algorithm, we were able to reach 56 participants by phone for an interview. Reasons for unsuccessful contacts with 16 participants were shortage of staff (*n* = 15) or administrative error (*n* = 1). For the remaining 80 participants, 24 were unreachable by phone and e-mail, or did not reply to the interview questions. As shown in Table 2, almost all of the 56 participants interviewed reported an increase in work stressors, while stressors outside work were reported by less than half of the sample. The relative importance (balance) of the work/private stressor areas showed a strong emphasis on work stressors, with 50 % rated as reporting work stressors only, 29 % as predominantly work stressors plus some private life stressors, 14 % as having roughly equal shares of work stressors and private life stressors, 4 % as having predominantly private life stressors plus some work stressors and 2 % as having private life stressors only. In addition, one participant denied any increase in stressor exposure at all.

**Table 2:** Frequencies and proportions of interviewer collected ratings of self-reported stressors among the 56 persons with sustained stress or exhaustion warning that were reached by telephone

Question Stress rating	Work stressors *n* (%)	Private life stressors *n* (%)
Substantially increased	21 (37)	5 (9)
Moderately increased	20 (36)	10 (18)
Slightly increased	13 (23)	11 (20)
Not increased	2 (4)	30 (54)

### Relations between personality dimensions and a LUCIE indication

The BFI scores, collected at baseline (T0), showed a difference in the group score distributions of Neuroticism and Agreeableness (ANOVA p’s <0.001) with small to medium effect sizes, but no group difference on Extraversion, Openness or Conscientiousness (Table 3). Post Hoc comparisons revealed that only the subgroups SWS-GS and EWS-S differed clearly from controls, by having higher scores on Neuroticism and lower scores on Agreeableness, although the subgroup SWS-AS also tended to have a higher score on Neuroticism, bordering on statistical significance (*p* = 0.054).

**Table 3:** Mean ratings on the Big Five personality inventory for subgroups with sustained LUCIE indication and controls

Personality dimension	SWS-AS (*n* = 15)		SWS-GS (*n* = 41)		EWS-S (*n* = 35)		CONTROLS (*n* = 723)		ANOVA	
	Mean	SD	Mean	SD	Mean	SD	Mean	SD	*p*	*η*_*p*_^*2*^
Neuroticism	2.63	.54	2.69**	.70	2.89**	.63	2.33	.59	<0.001	0.052
Extraversion	3.53	.80	3.55	.67	3.31	.77	3.59	.67	0.12	--
Openness	3.54	.46	3.44	.65	3.47	.67	3.41	.61	0.75	--
Agreeableness	4.04	.40	3.67**	.63	3.68**	.54	3.94	.43	<0.001	0.029
Conscientiousness	3.96	.51	4.01	.52	3.93	.46	3.94	.48	0.82	--

* *p* ≤0.05; **p ≤ 0.001, Post Hoc LSD test compared to controls

## Discussion

The present longitudinal study aimed to evaluate whether the newly developed LUCIE instrument could accurately detect individuals developing incipient exhaustion. Additionally, we examined whether changes in the work situation and/or the private life were present during the period of time leading up to a sustained stress or exhaustion warning in LUCIE. Our main hypothesis was that signs of incipient exhaustion should be primarily associated with a perceived increase in work stress.

### Principal findings

During the 3-year study period, 96 participants fulfilled the criteria for either a sustained stress warning (*n* = 64) or a sustained exhaustion warning (*n* = 36). The generally high KEDS scores at the time of fulfillment of sustained stress or exhaustion warning criteria seem to confirm the presence of slight but genuine ED symptoms. Moreover, participants who developed a sustained stress or exhaustion warning had higher Neuroticism scores at baseline than controls did. Increasing ratings of negative changes in private life and work life were also more common among the participants with LUCIE indications than among controls (58 % vs. 29 % and 87 % vs. 48 %, respectively). However, the free-text commentaries indicated that almost half of the ratings of negative changes within the private sphere in the LUCIE indication groups were based on work-stress-related *private life compromises.* In contrast, 87 % of the control group participants reported that traditional private burdens *unrelated to work* constituted all negative changes in private life. Among the 56 LUCIE indication participants interviewed, 79 % reported exclusively/predominantly work stressors, and only 6 % described a predominance of stressors in private life. Thus, the prospective stressor ratings from the websurvey and the retrospective interview data seem to provide a congruent picture with a strong emphasis on increased work stressor exposure.

### General discussion

Surprisingly, the transitions from normal LUCIE scores to sustained stress or exhaustion warnings were not *preceded* by reports of negative changes in the work situation. Instead, the prospective changes in LUCIE, and on the single work change item, appeared more or less simultaneously. One possible explanation might be that the time-frame for retrospective changes in the work situation question was slightly longer (for the past couple of months) than the corresponding time-frame in the LUCIE (last month). This implies that the participants might have projected their reflections on changes in their work situation for a period of several months back, while the LUCIE was replied to from a more short-term perspective, such as the recent 4 weeks. In addition, concrete negative work events might constitute more tangible or factual autobiographical facts in memory, while subtle stress signs might be harder to recall over several weeks. If so, negative changes in the work situation might in reality have preceded the increased LUCIE score with a month or two, although this remains a speculation.

To what extent changes in LUCIE scores can be attributed to a mere reinterpretation of the work situation (e.g., a stagnated poor work situation that gradually deprives the person of hope for future improvements and thus over time starts inducing negative outcome expectancies) or an actual change in the work conditions (e.g., increased workload, organizational problems etc.) cannot be disentangled by the quantitative data. However, the free-text commentaries revealed that participants with any LUCIE indication attributed negative changes at work mainly to increased workload and/or increased emotional or intellectual demands from the employer (including due to shortage of staff), reduced support from supervisor or colleagues, and organizational problems or negative organizational change (including conflicts *within* management). Such themes, or highly similar themes, have been shown to predict future mental health problems in terms of reduced mental well-being [16]. Conversely, the free-text responses indicating *positive* changes in the work situation often mirrored the thematic content of the negative changes; main positive themes were, for example, enriched decision latitude or more exciting/stimulating work tasks, improved support from supervisor and colleagues, and reduced workload (Additional file 2). However, reports on successful organizational change were scarce.

Many participants with sustained stress or sustained exhaustion warnings reported in their free-text commentaries that negative changes in *private life* concerned perceptions of work-to-family conflicts and deteriorating health due to excessive workload. When excluding such perceived “workload-causing-health-and-family-problems” themes, the frequency of negative reports in private life dropped substantially in the three groups with a sustained stress or exhaustion indication in LUCIE (from 58 to 32 %). The corresponding drop among the controls, reporting negative changes in private life, was negligible (from 29 to 26 %). Turning to reports of *positive* changes in the *work* situations, we observed that these reports occurred equally frequently among the three groups of participants with LUCIE indications as they did among controls (54 % vs. 48 % among controls), but that the (positive) theme of *voluntary reduction of work-hours in order to cope with life and family* was reported by 15 % in the LUCIE indication groups. This indicates that intentional reduction of workload was sometimes reported as an improved *private life* situation, though it in fact reflected an effect of reduced workload and would have been more correctly classified as a *work-related* positive change.

Another factor that may influence the development of sustained stress/exhaustion is variations in personal dispositions such as personality. The brief BFI inventory was completed at baseline (T0), that is, 2–10 quarters before the participants could fulfill any of the LUCIE indication algorithm criteria. Accordingly, most BFI ratings were made well ahead of the ratings that led to the fulfillment of sustained stress/exhaustion indication criteria. Interestingly, we observed that higher Neuroticism at baseline seems to be related to an increased risk of developing a sustained stress/exhaustion indication. This corresponds with findings from the initial cross-sectional validation study of the LUCIE, where participants with a LUCIE indication showed higher levels of Neuroticism [11]. In our previous study, we were unable to assess whether exhaustion-related psychological and physiological changes had already taken place and suggested as one explanation that the elevated levels of neuroticism might reflect an exhaustion-related personality change. Such an explanation would be reasonable in view of recent observations of increased neuroticism after exposure to bullying [17]. The results of the present prospective study, however, do seem to support the notion that neuroticism constitutes a vulnerability factor for developing stress symptoms, in accordance with results from crossectional studies [18].

### Strengths and limitations

One strength of the present study is the long observation time and the relatively frequent assessments (each quarter of the year). Other strengths are the fairly good compliance, yielding satisfactory response rates during the entire study period, and that we managed to assemble a cohort of persons with mainly higher education and (presumably) complex/demanding work tasks, and thus with an assumed higher likelihood of developing incipient exhaustion. Nonetheless, the participants were positively (self-)selected and the overall response rate into the study was low (18 %). Obviously this entails some limitations and warrants caution with regard to generalizing the results to other contexts and populations.

The validity and reliability of the two newly designed questions on changes in work and family life are unknown. Yet the vast majority of participants provided free-text commentaries that concurred with the single-item questions concerning work-related stressors. The free-text commentaries also showed that many ratings of negative changes in private life in fact represented a feeling of being worn out by work, or having a work situation that meant reduced time and energy for desired private life activities. In parallel, reducing work hours – on the initiative of the employee – was sometimes reported as a positive change in the *work* situation. Thus, affirmative replies to positive work/private changes seemed to be inflated by reporting several work-related effects as belonging to private life. This observation highlights the fact that individuals cannot easily separate “work” and “private” factors in the way that researchers may assume. This indicates that obtaining supplementary qualitative information on ratings, as was done in the present study, may be essential to properly classifying the contents of ratings.

Why we sometimes collapsed the three groups *SWS-AS*, *SWS-GS* and the *EWS-S* into one single group may also be called into question. The reason for this procedure was primarily a matter of feasibility. Because the separate subgroups consisted of fairly few participants, and because the underlying difference between the SWS subgroups and the EWS subgroup is primarily a matter of intensity (and not of a qualitatively different symptomatology), we decided to analyze all three LUCIE indication groups as a whole, in order to provide a reasonable general trend in the reported changes in life associated with increasing LUCIE scores. In addition, the ratings of changes in work/private life and the associated free-text themes were indistinguishable across groups.

The use of only *one* preceding quarter (the baseline questionnaire) for detecting the initial set of LUCIE indication participants in the third quarter (T2) might also be discussed, as it might have led to less robust detection of sustained stress and exhaustion warning in LUCIE (possibly due to a less trustworthy “normal” LUCIE score at baseline). This simplification was, however, justified by our ambition to fine tune our study routines and to target as many LUCIE indication cases as possible. In any event, and if anything, this decision is only likely to have led to the identification of milder/diffuse cases and, if so, would have weakened the reported group differences.

Finally, we should also consider that an individual with stress symptoms might find it easier to attribute symptoms to the work situation than to a personal inability to deal with the hassles of daily life, including work life. Indeed, attribution of health problems to factors outside the individual’s control is commonly encountered in, for example, medically unexplained symptoms [19]. Furthermore, in Sweden and many other countries, the attribution of health problems to working conditions follows a long labor union tradition focused on humanizing work conditions [20, 21]. Thus, there might exist a more or less ‘unconscious’ bias in attributing health problems to work, instead of seeking clues in personal attitudes, lifestyle, physical shortcomings and other non-work-related factors. It is possible that such phenomena have to some extent inflated the participants’ rating of work stressors in this study, for example concerning the data derived from the telephone interview in which the opening question concerned potential work stressors. We cannot deny the potential importance of this aspect, although its possible confounding effect is probably shared with other studies in the area of work stress research.

## Conclusion

The present study, which prospectively evaluated LUCIE, a new instrument designed for detection of the prodromal stages of exhaustion, showed that sustained stress or exhaustion warnings in LUCIE were confirmed by subtle indications of exhaustion disorder in the Karolinska Exhaustion Disorder Scale. In addition, indications in LUCIE corresponded well with a recent experience of negative changes in the work situation. To conclude, the LUCIE seems to be a suitable clinical tool for detecting incipient exhaustion. It is our hope that LUCIE can be implemented in an effort to prevent the development of full-blown exhaustion disorder or any similar condition possibly resulting from a long-term stressful work situation.

## Additional files

Additional file 1: Computation algorithms for the LUCIE scales SWS and EWS. (DOCX 75 kb) Additional file 2: Frequencies of main themes of positive/negative changes in the work situation and private life, derived from free-text commentaries among participants targeted by the LUCIE algorithms. (DOCX 82 kb)

## Abbreviations

BFI: Big five inventory
ED: Exhaustion disorder
EWS: Exhaustion warning scale
EWS-S: Onset to sustained exhaustion warning
ICD-10: International Statistical Classification of Diseases and Related Health Problems - 10th Revision
KEDS: Karolinska exhaustion disorder scale
LUCIE: Lund University Checklist for Incipient Exhaustion
NBHW: Swedish National Board of Health and Welfare
SWS: Stress warning scale
SWS-AS: Abrupt onset to sustained stress warning
SWS-GS: Gradual onset to sustained stress warning

## References

[1] Joyce S, Modini M, Christensen H, Mykletun A, Bryant R, Mitchell PB (2016). Workplace interventions for common mental disorders: a systematic meta-review. Psychol Med.

[2] Hensing G, Wahlstrom R (2004). Swedish Council on Technology Assessment in Health Care (SBU). Chapter 7. Sickness absence and psychiatric disorders. Scand J Public Health Suppl.

[3] Rai D, Kosidou K, Lundberg M, Araya R, Lewis G, Magnusson C (2012). Psychological distress and risk of long-term disability: population-based longitudinal study. J Epidemiol Community Health.

[4] Swedish Social Insurance Agency (2015). Sjukskrivningar 60 dagar eller längre. En beskrivning av sjukskrivna åren 1999-2014 efter kön, ålder, arbetsmarknadsstatus, yrke, sjukskrivningslängd och diagnospanorama. (In Swedish). Socialförsäkringsrapport 2015:1.

[5] Swedish National Board of Health and Welfare (2003). Exhaustion Disorder (In Swedish; Utmattningssyndrom).

[6] Maslach C, Schaufeli W, Leiter M (2001). Job Burnout. Annu Rev Psychol.

[7] Peterson U, Bergström G, Samuelsson M, Åsberg M, Nygren A (2008). Reflecting peer-support groups in the prevention of stress and burnout: randomized controlled trial. J Adv Nurs.

[8] van der Klink JJ, Blonk RW, Schene AH, van Dijk FJ (2003). Reducing long term sickness absence by an activating intervention in adjustment disorders: a cluster randomised controlled design. Occup Environ Med.

[9] Karlson B, Jönsson P, Österberg K (2014). Long-term stability of return to work after a workplace-oriented intervention for patients on sick leave for burnout. BMC Public Health.

[10] Karlson B, Jönsson P, Pålsson B, Åbjörnsson G, Malmberg B, Larsson B (2010). Return to work after a workplace-oriented intervention for patients on sick-leave for burnout--a prospective controlled study. BMC Public Health.

[11] Persson R, Österberg K, Viborg N, Jönsson P, Tenenbaum A (2016). The Lund University Checklist for Incipient Exhaustion – a cross-sectional comparison of a new instrument with similar contemporary tools. BMC Public Health.

[12] Rosvall M, Grahn M, Modén B, Merlo J (2009). Hälsoförhållanden i Skåne. Folkhälsoenkät Skåne 2008. (In Swedish).

[13] Besér A, Sorjonen K, Wahlberg K, Peterson U, Nygren Å, Åsberg M (2014). Construction and evaluation of a self rating scale for stress-induced exhaustion disorder, the Karolinska Exhaustion Disorder Scale. Scand J Psychol.

[14] John OP, Srivastava S, Pervin LA, Oliver P (1999). The Big Five Trait Taxonomy. History, measurement, and theoretical perspectives. Handbook of Personality: Theory and Research.

[15] McCrae RR, Costa PT, Pervin LA, Oliver P (1999). A five-factor theory of personality. Handbook of personality: Theory and research.

[16] Nieuwenhuijsen K, Franche RL, van Dijk FJH (2010). Work functioning measurement: tools for occupational mental health research. J Occup Environ Med.

[17] Persson R, Hogh A, Grynderup MB, Willert MV, Gullander M, et al. Relationship Between Changes in Workplace Bullying Status and the Reporting of Personality Characteristics. J Occup Environ Med. 2016, in press. 10.1097/JOM.0000000000000822 27454394

[18] Garbarino S, Chiorri C, Magnavita N (2014). Personality traits of the five-factor model are associated with work-related stress in special force police officers. Int Arch Occup Environ Health.

[19] Brown RJ (2007). Introduction to the special issue on medically unexplained symptoms: background and future directions. Clin Psychol Rev.

[20] Vaananen A, Anttila E, Turtiainen J, Varje P (2012). Formulation of work stress in 1960–2000: analysis of scientific works from the perspective of historical sociology. Soc Sci Med.

[21] Johansson J, Abrahamsson L (2009). The good work--a Swedish trade union vision in the shadow of lean production. Appl Ergon.

