# Supplementary material for: The Lund University Checklist for Incipient Exhaustion: a prospective validation of the onset of sustained stress and exhaustion warnings
Source: BMC Public Health. 2016 Sep 29;16:1025. doi: 10.1186/s12889-016-3720-7 (PMC5043621; doi:10.1186/s12889-016-3720-7)
Supplement: Additional file 1: — Computation algorithms for the LUCIE scales SWS and EWS. (DOCX 75 kb) [file 12889_2016_3720_MOESM1_ESM.docx]

**Additional file 1. Computation algorithms for the LUCIE scales SWS and EWS**

***(A) SWS=the Stress Warning Scale*** is aimed at capturing milder long-term stress symptomatology in order to detect *pre-stages* of exhaustion. To construct the SWS scale, item scores for 26 items (excluding item 10 and 13) were dichotomized in the middle of the response scale (between the scale steps “somewhat”[2] and “quite a bit”[3]), which means that a response on scale step 3 (or 4) was scored as “1” and a response on scale step 1 or 2 was scored as “0.” The means of the summed scores within each problem area/dimension (A-F) were calculated separately, resulting in six mean values for the six problem areas/dimensions, each with a possible scoring range between 0 and 1. These six mean scores were transformed into a total score by computing the mean of the six area/dimension means, leading to an equal weighting of each problem area/dimension (A-F). For convenience, the scoring range was multiplied by a factor of 100, providing a final SWS score that ranged from 0 to 100.

*Calculation of the SWS scale score:*

Step 1 - recoding of SWS items: All item scores 1 and 2 are recoded as 0 (zero), and all item scores of 3 and 4 are recoded as 1.

Step 2 - computation of SWS: SWS = 100 x [(Sum of scores on Item 1 to 3)/3) + ((Sum of scores of Item 4 to 7)/4) + ((Sum of scores of Item 8 and 9)/2) +((Sum of scores on Item 11, 12 and 14)/3) + ((Sum of scores Item 15 to 17)/3) + ((Sum of scores on Item 18 to 28)/11)] /6.

***(B) EWS=the Exhaustion Warning Scale*** is intended to differentiate only ED from both of the other groups (having no or mild stress symptomatology); it was optimized by dichotomizing the responses to 28 items around the two highest scale steps (between “quite a bit” [3] and “very much” [4]), while the responses to 2 items were dichotomized in the midpoint of the scale (between the scale steps “somewhat”[2] and “quite a bit”[3]). A response on step 4 was scored as “1” and a response on step 3 or below was scored as “0”, except for items 10 and 13 for which a response on scale step 3 (or 4) was scored as “1” and a response on scale step 1 or 2 was scored as “0.” Similar to the procedure for the SWS, the means of the summed scores within each problem area/dimension (A-F) were calculated separately, resulting in six mean values for the six problem areas/dimensions, each with a possible scoring range between 0 and 1.

*Calculation of the EWS scale score:*

Step 1 - recoding of EWS items: Items scores 1 to 3 are recoded as 0 (zero) and items scores of 4 are recoded as 1. On items 10 (control of work duties) and 13 (less able to make decisions), the scores 1 and 2 are recoded as 0 (zero) and items scores of 3 and 4 are recoded as 1.

Step 2 - computation of EWS: EWS = 100 x [(Sum of scores of Items 1 to 3)/3) + ((Sum of scores of Items 4 to 7)/4) + ((Sum of scores of Items 8 and 9)/2) +((Sum of scores on Items 10 to 14)/5) + ((Sum of scores Item 15 to 17)/3) + ((Sum of scores Item 18 to 28)/11)] /6.

Note that in cases of missing responses on any item in SWS or EWS, the divisor for that subgroup of items is reduced to reflect the mean value of the items that are responded to.
