# Supplementary material for: The Lund University Checklist for Incipient Exhaustion: a prospective validation of the onset of sustained stress and exhaustion warnings
Source: BMC Public Health. 2016 Sep 29;16:1025. doi: 10.1186/s12889-016-3720-7 (PMC5043621; doi:10.1186/s12889-016-3720-7)
Supplement: Additional file 2: — Frequencies of main themes of positive/negative changes in the work situation and private life, derived from free-text commentaries among participants targeted by the LUCIE algorithms. (DOCX 82 kb) [file 12889_2016_3720_MOESM2_ESM.docx]

**Additional file 2. Frequencies of main themes of positive/negative changes in the work situation and private life, derived from free-text commentaries among participants targeted by the LUCIE algorithms**

**Table 2:1. Frequencies of main themes of *negative* changes in the work situation derived from free-text commentaries among participants targeted by the LUCIE algorithms (n=77).**

| **Main theme** | **n** | **%** |
| --- | --- | --- |
| Increased workload and/or increased emotional or intellectual demands from employer, including shortage of staff | 44 | 57 |
| Reduced support from supervisor or colleagues | 18 | 23 |
| Organizational problems or negative organizational change, including conflicts within management | 10 | 13 |
| “Job Stress” | 11 | 14 |
| Job insecurity | 9 | 12 |
| Reduced decision latitude or quality of work contents | 8 | 10 |
| Less comfortable (or longer) working hours | 7 | 9 |
| Reduced reward (salary, acknowledgment) | 6 | 8 |
| Injustice on the part of supervisors, including unfairness in work task assignment | 5 | 7 |
| Unsuccessful move to new employer | 4 | 5 |
| Conflicts with colleagues or supervisors | 4 | 5 |
| Reduced personal work engagement | 3 | 4 |
| Multiple worksites/multiple roles | 2 | 3 |

Note. The rates above show the number of individuals reporting a change within each category separately. Thus, any one individual could report changes across several categories.

**Table 2:2. Frequencies of main themes of *positive* changes in the work situation derived from free-text commentaries among participants targeted by the LUCIE algorithms (n=40).**

| **Main theme** | **n** | **%** |
| --- | --- | --- |
| Enriched decision latitude or more exciting/stimulating work tasks | 12 | 30 |
| Improved support from supervisor, colleagues or through group intervention | 10 | 25 |
| Successful move to new employer | 7 | 18 |
| Reduced workload and/or decreasing emotional or intellectual demands from supervisors, including reduced shortage of staff | 7 | 18 |
| Improved working hours | 6 | 15 |
| Increased reward (salary, acknowledgment, education) | 3 | 8 |
| Less job insecurity | 2 | 5 |
| Successful organizational change | 2 | 5 |
| Reduced stress | 2 | 5 |
| Education in stress management techniques | 1 | 3 |
| Improved work space | 1 | 3 |

Note. The rates above show the number of individuals reporting a change within each category separately. Thus, any one individual could report changes across several categories.

**Table 2:3. Frequencies of main themes of *negative* changes in the private life situation derived from free-text commentaries (n=46).**

| **Main theme** | **n** | **%** |
| --- | --- | --- |
| Work-family conflict (lack of time/energy) | 22 | 48 |
| Worn out (fatigue, exhaustion) | 11 | 24 |
| Relational problems in family | 7 | 15 |
| Serious illness/death of close relative/close friend | 7 | 15 |
| Worries for children | 4 | 9 |
| Caregiving for elderly next of kin | 4 | 9 |
| Negative divorce/separation | 2 | 4 |
| Being fired or spouse fired | 2 | 4 |
| Child custody dispute | 1 | 2 |
| Economic stress | 1 | 2 |
| Neglected lifestyle/physical fitness | 1 | 2 |
| Longer commuting | 1 | 2 |
| Other specific burden | 1 | 2 |

Note. The rates above show the number of individuals reporting a change within each category separately. Thus, any one individual could report changes across several categories.

**Table 2:4. Frequencies of main themes of *positive* changes in the private life situation derived from free-text commentaries (n=43).**

| **Main theme** | **n** | **%** |
| --- | --- | --- |
| Improved family relations or family situation | 16 | 37 |
| Voluntary reduction in work hours to cope with life/spending more time with family | 6 | 14 |
| Parenthood/pregnancy (incl. grandchildren) | 5 | 12 |
| Falling in love/new relation | 4 | 9 |
| Improved energy/vitality | 4 | 9 |
| Positive divorce/separation | 3 | 7 |
| Relaxing vacation | 3 | 7 |
| New hobby or new friends | 3 | 7 |
| Improved dwellings | 3 | 7 |
| Improved lifestyle | 3 | 7 |
| Restructured life plans/attitudes | 2 | 5 |
| Other unspecific improvement | 2 | 5 |
| Shorter commuting | 1 | 2 |

Note. The rates above show the number of individuals reporting a change within each category separately. Thus, any one individual could report changes across several categories.
